# Novel Treatments, Approaches, Prevention Strategies, and Insights in Pediatric, Adolescent, and Gynecological Endocrinology

**DOI:** 10.3390/children12101358

**Published:** 2025-10-09

**Authors:** Dimitrios T. Papadimitriou

**Affiliations:** Neonatal-Pediatric-Adolescent Endocrinology & Diabetes, Department of Pediatrics, University General Hospital of Larissa, 413 34 Larissa, Greece; dtpapad@uth.gr

## 1. Introduction

The fields of neonatal, pediatric, and adolescent gynecological endocrinology are undergoing a transformative shift. With the tremendous advances recently made in genetics and precision medicine [1], imaging, and molecular biology, a deeper understanding of the maternal as well as the embryonic hormonal milieu—taking into account the startling consequences of chronic stress [2], as well as environmental factors and the role of endocrine-disrupting mechanisms [3]—has enabled clinicians and researchers to address the diverse spectrum of endocrine disorders with a more holistic and hopefully genuine Hippocratic approach [4]. This topical collection in *Children* (ISSN 2227-9067) has brought together contributions that highlight the rich complexity and compelling challenges of this field. These studies not only enhance clinical knowledge but also encourage a multidisciplinary, forward-thinking approach to patient care with direct impacts on child care and safety, global health, and the emerging worldwide problem of infertility recognized by the World Health Organization [5].

## 2. Growth, Bone Health, and Hormonal Axes

Accurate growth monitoring remains a cornerstone of pediatric endocrinology. A comprehensive mixed longitudinal study of Greek schoolchildren offers new height velocity charts and defines the milestones of the adolescent growth spurt (Contribution 1). By identifying critical time points such as take-off and peak height velocity, these data provide practical value in detecting abnormalities in pubertal timing and development. Interestingly, this study confirms that growth acceleration typically precedes overt signs of puberty, supporting the hypothesis that hormonal triggers such as IGF-1 and estradiol initiate pubertal changes well in advance.

In a related investigation, follistatin, a hormone involved in the activin–follistatin–inhibin system, was associated with bone mineral density in lean adolescent girls with high physical activity levels (Contribution 2). While follistatin accounted for only a small portion of the variance in bone density, its independent predictive value for lumbar spine bone mineral density—a region rich in trabecular bone—merits further exploration, especially in the context of bone health promotion in physically active youth.

The interplay of growth hormone, parathyroid hormone (PTH), and vitamin D remains a focus of endocrine research. A four-year longitudinal study of children treated with recombinant human growth hormone showed a stable vitamin D status but a persistent rise in PTH levels (Contribution 3). Despite no overt biochemical deficiencies, these findings suggest that GH therapy may subtly alter calcium–phosphate metabolism, emphasizing the need for regular monitoring.

## 3. Genetic Disorders, Molecular Diagnostics, and Cancer

Genetic syndromes are often at the heart of pediatric endocrine pathologies. A novel TP53 gene variant causing Li–Fraumeni syndrome demonstrates the critical role of whole-genome sequencing in identifying cancer predisposition in young patients (Contribution 4). This particular case allowed for early detection of multiple tumors in both the index case and her relatives, reinforcing the importance of genetic counseling and surveillance.

Another report detailed a rare Ala871Glu mutation in the androgen receptor gene in a girl with Complete Androgen Insensitivity Syndrome (Contribution 5). Genetic confirmation helped guide management, including gonadectomy and hormone replacement, and expanded the mutation spectrum associated with this rare disorder.

Meanwhile, a systematic review on Mayer–Rokitansky–Küster–Hauser (MRKH) syndrome compiled evidence on chromosomal abnormalities and mutations of genes such as WNT4, LHX1, and HOXA (Contribution 6). The review facilitates a more targeted diagnostic approach, especially for patients with syndromic features or familial clustering.

The diagnostic journey of a 1-month-old infant with Type Ia pseudohypoparathyroidism illustrates the value of vigilant physical examination and genetic testing (Contribution 7). Despite the infant having normal biochemical values at the time of diagnosis, phenotypic findings and early GNAS mutation analysis enabled prompt recognition of the condition, underscoring the value of integrated diagnostic strategies.

## 4. Endocrine Aspects of Oncology and Fertility Preservation

The intersection between cancer therapy and endocrinology presents unique clinical dilemmas. Ovarian tissue cryopreservation has emerged as a promising fertility-preserving option for children and adolescents undergoing gonadotoxic treatments (Contribution 8). Its benefits—especially in prepubertal girls—include the avoidance of hormonal stimulation and the preservation of high-quality primordial follicles. Nonetheless, concerns about malignant cell reimplantation remain. Molecular and immunohistochemical screening techniques may enhance safety and effectiveness moving forward.

Fetal thyroid development is another critical area, particularly when influenced by maternal factors. A narrative review highlights the impact of thyrotropin-releasing hormone (TRH) and antithyroid drugs during gestation (Contribution 9). With TRH now known to be produced by extrahypothalamic tissues and capable of crossing the placenta, it may play a more prominent role in fetal endocrine development than previously appreciated. The findings of this study stress the importance of precise maternal endocrine management during pregnancy.

Thyroid malignancies in children are rare, yet their incidence is reportedly increasing. A Romanian study on pediatric thyroid microcarcinoma observed a significant number of cases in adolescents, possibly linked to environmental exposures such as iodine deficiency and past nuclear incidents (Contribution 10). These findings advocate harmonized treatment protocols and international collaboration, possibly through platforms like Endo-ERN.

## 5. Early-Life Determinants of Endocrine and Metabolic Disorders

A literature review on early-life risk factors for breast anomalies strengthens the link between childhood exposures—diet, radiation, and BMI—and later development of proliferative breast disease (Contribution 11). This review supports a life-course model of prevention, urging pediatricians to integrate risk-reducing strategies early in life.

Metabolic health in childhood remains an area of global concern. A study from Spain examined prepubertal children with obesity who exhibited metabolically healthy profiles (Contribution 12). After 24 months of following the Mediterranean diet and participating in a physical activity intervention, improvements were noted in inflammatory markers such as CRP and IL-6. However, the unexpected worsening of their adipokine profiles (lower adiponectin and higher resistin) calls for deeper investigation into how metabolic health is defined and monitored in young populations.

A novel prospective study will explore possible correlations between spinal dysraphisms and polycystic ovary syndrome (PCOS), given the presence of common risk factors and similar alterations in homocysteine and inositol metabolism (Contribution 13).

## 6. Neuroendocrine and Immunoendocrine Interfaces

The complex interplay between hormonal and neural development is highlighted in a neuroimaging study of girls with idiopathic central precocious puberty (Contribution 14). The study revealed increased volumes in several gray and white matter regions compared to age-matched peers, with some volumes correlating positively with LH levels. These findings suggest that brain maturation in ICPP may follow an accelerated, yet physiologically typical, trajectory—potentially influencing cognitive or behavioral development.

Hormonal effects on immune function are explored in a review on perimenstrual asthma, a condition marked by cyclical worsening of asthma symptoms around menstruation (Contribution 15). The paper explores novel hormonal therapies and even microbial-directed strategies, reflecting the need for more adolescent-focused asthma management guidelines that consider endocrine fluctuations.

A parallel concern is the management of neonatal inflammation. A hypothesis proposed in another article considers partial blocking of cell adhesion molecule function as a means to mitigate perinatal inflammation, particularly in neonates with leukocyte adhesion deficiency (Contribution 16). The theoretical use of antibody or peptide-based therapy could represent a new frontier in neonatal immunomodulation.

## 7. Emerging Genetic Insights with Social Impact

A compelling case describes multiple fetal fractures in a newborn later found to carry a homozygous pathogenic mutation in the CCDC134 gene (Contribution 17). The mother’s diagnosis of Ehlers–Danlos syndrome further complicated the diagnostic picture. This case demonstrates how detailed genetic analyses can prevent misdiagnoses, such as nonaccidental trauma, and guide appropriate care pathways.

## 8. Conclusions

This Topical Collection of *Children* captures the dynamic and interdisciplinary nature of pediatric endocrinology. From new genetic discoveries and growth assessment tools to novel fertility preservation strategies and neurodevelopmental insights, the included articles exemplify how precision medicine is reshaping pediatric endocrine care. As we continue to refine diagnostic algorithms, therapeutic approaches, and preventive strategies, ongoing international collaboration will be key in ensuring all children receive care informed by the latest scientific advancements.
